# Prevention of Unhealthy Weight, Disordered Eating, and Poor Body Image in Children. Perspectives From Norwegian Parents and Healthcare Professionals

**DOI:** 10.3389/fpsyt.2022.895781

**Published:** 2022-04-27

**Authors:** Charlotte Fiskum, Åshild Riiber, Trine Tetlie Eik-Nes

**Affiliations:** ^1^Department of Psychology, Faculty of Social and Educational Sciences, Norwegian University of Science and Technology, Trondheim, Norway; ^2^Healthy Life Centre (Friskliv og mestring), Orkanger, Norway; ^3^Department of Neuromedicine and Movement Science, Faculty of Medicine and Health Sciences, Norwegian University of Science and Technology, Trondheim, Norway; ^4^Stjørdal Community Mental Health Centre, Levanger Hospital, Nord-Trøndelag Hospital Trust, Levanger, Norway

**Keywords:** childhood obesity, body dissatisfaction, family-based interventions, parent-centered, parent engagement, stress-sensitive

## Abstract

**Background:**

Childhood obesity (ChO) and eating disorders are on the rise, with concerning effects on health. Early prevention is essential as interventions after problems arise are costly and with a low success rate. In Norway, prevention of ChO has been largely weight-centered, without desired effects. Confident Body, Confident Child (CBCC) is a universal program aimed at preventing ChO, disturbed eating, and body image problems through a health-centered intervention for parents of children between 2 and 6 years. The current study is part of a cultural adaptation and translation of CBCC into Norwegian.

**Methods:**

Focus groups with parents (*n* = 16) and professionals (*n* = 11) were held around healthy eating, activity, and body image, with an emphasis on possible barriers for prevention as well as approaches considered helpful. The interviews were analyzed using interpretative phenomenological analysis.

**Results:**

Parents and professionals described parental stress connected to high standards, conflicting information, and parental comparison. A narrowing sense of normality around healthy living was described with little flexibility resulting in “all-or-nothing” thinking. Parents were anxious to say or do the wrong thing when regulating children's food intake and when faced with comments about appearance. Parents and professionals described parental concern around children not eating enough, and professionals described an increase in parents using food as regulation. Both parents and professionals expressed that having a child with overweight was tied to a sense of failure and shame. Interventions related to overweight seemed to increase stress and shame, further complicating follow-up. As an alternative, parents and professionals expressed a desire for interventions with normalizing information around “good-enough” parenting related to food and weight.

**Discussion:**

The described fear of doing something wrong and lack of flexibility is interpreted within a stress-sensitive understanding, where stress and shame can influence parents toward mobilizing action or disengagement, presenting as dichotomous behaviors of “all-or-nothing”.

**Conclusion:**

Interventions that can normalize parental concerns in a non-moralizing way may reduce stress and shame. CBCC addresses all the major concerns raised in this study, providing parents with evidence-based information they can implement into everyday life. The Norwegian cultural adaptation added extra emphasis on normalization and shame-reduction.

## Introduction

The recent decades have seen a significant increase in obesity in children and adolescents (1, 2). Childhood obesity (ChO) at as young as 5 years of age predicts later obesity (3), children suffering from ChO show a five-fold increase in risk for adult obesity compared to children without ChO (4), and more than 60% of children with ChO will grow up to become adults with obesity (5). This increase is concerning from both individual and societal health perspectives (6). Serious health issues from ChO can arise early, with adverse effects to virtually every organ in the body (7, 8). The steep increase of chronic non-communicable diseases like cardiovascular disease, diabetes, cancer, and mental health disorders may, at least in part, be due to the increase of obesity in society and in childhood (7, 9).

An increasing number of studies show that treatment of ChO is complicated, providing mixed results (10, 11) and mostly small, short-term reductions in weight and body mass index (BMI) in children and adolescents (11–14). Weight reduction in ChO may also have only minor positive effects on cardiometabolic risk profiles, particularly in boys (15). Hence, more effective preventative measures are needed to prevent ChO. Preventative efforts should likely start in early childhood, as the probability of developing ChO increases after the age of two (16). In addition, early interventions are generally considered superior to interventions later in life regarding unhealthy weight development and disordered eating (1, 17–19).

Caregivers represent the single most important point of influence in early childhood. A review of preventative interventions for ChO concluded that interventions should approach ChO from a health-centered parental perspective rather than an individual weight-centric perspective (20). Notably, the style of parenting and feeding practices were found to affect both lifestyle habits and help the child become aware of internal cues of hunger or satiety, which are essential for regulating eating in a society surrounded by external food-related cues (20). Family responsibilities (such as keeping a healthy lifestyle) can cause significant parental stress (21, 22). As living with elevated stress levels can impact brain responses to food in favor of high calorie “comfort” foods (23) and affect family dietary quality and parental feeding practices negatively (24, 25), as well as complicate follow up (26) parental stress is of importance when considering ChO. To understand people's behaviors when faced with stressful situations or demands, it is helpful to understand common defensive reactions. Research shows that exposure to a stressor or threat often causes people to respond with either active engagement or passive withdrawal and disengagement (27–29). In terms of healthy living, active engagement could include seeking out information and controlling factors such as sugar intake. At the same time, passive avoidance could entail not seeking out information, in addition to not engaging in, or (seemingly) caring about, health-promoting behaviors. As the perceived stress increases or the resources and ability to cope decreases, more active, solution-focused strategies will often give way to increasingly passive and avoidant strategies and disengagement (27, 29, 30). In line with this, family disengagement and the presence of more socio-economic stressors have been identified as barriers to interventions targeting ChO (31). Thus, programs that can lower parental stress and increase engagement around healthy eating and body image may positively impact parental practices. However, most current interventions are centered on child weight and weight reduction (32, 33) and do not consider family or parental stress.

In addition, interventions rarely consider the possible adverse effects of individual weight-centric interventions on child body image (34). The growing number of children and adolescents who struggle with difficult thoughts and feelings regarding their weight and body image makes the lack of interventions for ChO that promote a healthy body image concerning. Difficulties with body image can increase the risk of later unhealthy eating patterns, weight problems, inactivity, and eating disorders (ED) (35–37). Like ChO, ED has a high lifetime morbidity and mortality rate and is hard to treat (38). Eating disorders are becoming increasingly prevalent, particularly among younger people and children (38, 39). Therefore, the prevention of ED is a significant public health concern due to the high incidence of medical complications (38). ChO and ED share common risk factors (40), and weight-focused anti-obesity efforts can increase the risk of ED (38). For example, a prospective study on early onset ED showed that body dissatisfaction was the most consistent predictor (37). Therefore, a simultaneous focus on healthy body image and disordered eating in the prevention of ChO is likely important to avoid the onset of ED. Hence, early prevention targeting weight problems, disordered eating, and negative body image in children while considering parental stress is of public health interest.

There is currently no early prevention intervention in Norway for parents or professionals related to early healthy eating practices and body image in children. In Norway, children are examined regularly from birth to adolescence following national preventive health program guidelines. Weight and height measurements are done at three time points, primary school entry at age 5–6 years, third grade, and eighth grade. All children with underweight, overweight, or obesity based on International Obesity Task Force (IOTF)-recommended definitions (41, 42) are offered follow-up or guidance from primary care health professionals based on national guidelines. The guidance provided for ChO or underweight is in Norway mainly centered on weighing, nutritional advice, and exercise and has shown relatively weak weight effects (43). It has also been shown that parents experience feedback on their child's weight status differently (44), with some parents refusing further follow-up.

Confident Body, Confident Child (CBCC) (19) is a parent prevention program developed in Australia from research evidence (32) and informed by consultation with parents (45) and experts (46). CBCC is a universal prevention resource aimed at preventing ChO, disordered eating, and body image disturbances. CBCC provides easy-to-implement parenting strategies to promote body satisfaction, healthy eating, and activity in 2–6-year-old children. CBCC consists of a 4-hour manualized workshop centered on psychoeducation, group discussions, and activities, also providing parents with a booklet, poster, and a website. The workshop and parent booklet provides facts around why health-promoting parental practices are important, followed by examples of how to perform the practices in everyday life, including challenging situations likely to cause stress and less reflective parental behaviors. In addition, the CBCC workshop format can help normalize everyday parent worries and practices in a group setting. The workshop is delivered over 2 weeks, allowing for a practice period. Parents are provided with homework and simple games and tasks centered on enjoying food, food preparation, and family meals, and positive and curious reflection around body image for the 2-week practice period. Parents are also provided with a program booklet and a poster summarizing the most important principles and parental skills. After the 2 weeks, the parental group meets with the counselor again for another session, starting with a discussion of their experiences and challenges over the 2 weeks to further consolidate and strengthen parental skills.

CBCC can effectively enhance parent and child-outcomes in randomized controlled trials (19, 47, 48), particularly when delivered to participants *via* the parent workshop. A recent study of CBCC suggests that the intervention may promote healthy eating patterns and child body image up to 18 months after parents received the intervention (48). CBCC represents an evidence-based resource for working preventatively with ChO, disordered eating, and body image simultaneously and can be implemented into primary care for universal prevention. For more vulnerable parents or families with fewer resources, CBCC can also be used in conjunction with individual consultations.

This study was done in preparation for a Norwegian translation and cultural adaptation of CBCC. To culturally adapt CBCC to Norwegian social and family surroundings, we explored parental and professional perspectives and concerns related to weight, eating behaviors, activity, screen time, and body image in three parental focus groups and three professional focus groups from urban and rural areas of Norway. In addition, the study explored parents' and professionals' opinions and experiences of approaches considered helpful or unhelpful to them. The paper presents perspectives on (1) challenges and concerns and (2) possible solutions for preventing ChO and body dissatisfaction. Possible solutions are discussed within a stress-informed perspective (27), where reactions to stress are seen as moving along a continuum from active engagement to passive disengagement, and interventions that can lower stress are essential (27, 29, 49).

## Materials and Methods

### Ethics

The project was reviewed by the regional committee for research ethics and the Norwegian Center for Research Data (NSD) and complied with the Helsinki declaration (50). All participants were informed of the study protocol before inclusion and gave written consent. As some parents could find body and weight-related questions stressful, the participants were provided with contacts for free counseling services regarding ED and mental health. Parents were also encouraged to speak to research staff members if they felt distressed.

### Recruitment and Participants

Primary healthcare services in Orkland (rural) and Trondheim (urban) municipalities working with weight or inactivity were invited to participate. Participants were recruited for two different focus groups: (1) for healthcare personnel or for (2) parents of children in preschool (age 2–6 years old). To recruit parents, information statements and consent forms were distributed in places frequently visited by parents and children in the two municipalities, such as childcare centers/kindergartens and schools. In addition, information about the project was posted on social media. Exclusion criteria was not being able to understand and speak Norwegian well enough to participate in a focus group.

### Inclusion Criteria

Participants in the parental focus groups had to be above 18 years old and have at least one child between 2 and 6 years of age. They also had to have a good comprehension of Norwegian. Participants in the professional focus groups had to have at least a bachelor's degree and experience working with children or families with weight problems or inactivity in a relevant position in primary care, such as public health nurses, physiotherapists, or occupational therapists. They also had to speak and understand Norwegian well. Exclusion criteria was not being able to understand and speak Norwegian well enough to participate in a focus group.

### Subjects

#### The Professional Groups

Eleven professionals participated across three focus groups for health care personnel, including three physiotherapists, one nutritional counselor (master's degree in food science), one midwife, and six public health nurses. All were female. Five professionals worked in a rural area (Orkland), the rest in an urban area (Trondheim). In focus group 1 (urban) 4 professionals participated, 2 professionals participated in focus group 2 (urban) and 5 professionals participated in the final, rural group. Their professional experience as primary care workers ranged from 9 to 30 years with a mean experience of 19 years. All the professionals had broad experience working with ChO and lifestyle chances (ranging from ~9 to 30 years), but this was harder to quantify in absolute terms for some participants, due to internal organization of their services.

#### The Parent Groups

Sixteen parents participated across three focus groups, including fifteen mothers and one father. Five parents lived in a rural area, the rest in an urban area, all were from the Norwegian majority culture. In focus group 1 (urban) 6 parents participated, 5 parents participated in focus group 2 (urban) and 5 parents participated in the final, rural group. All the parents gave demographic information. Fourteen parents were married or living with a partner, two parents lived alone. Their ages ranged from 30 to 38 years, they had between one and three children ranging from 0 to 7 years (mean age 3 years). Two parents had completed high school/vocational school as their highest level of education, six had completed a bachelor level education and eight had completed a master's degree.

### Interviews and Focus Groups

A semi-structured interview guide with 11 questions was used for all focus groups and expanded on an interview guide used previously (45) in an Australian setting. The questions were designed to elicit information about what parents and professionals understand by the terms “healthy eating, healthy physical activity, and body image”, what resources parents and professionals know of and use to learn about these topics, what they like and do not like about these resources, and what they find concerning or worrying around healthy eating, activity, and body image in children. The interview also asked what would help increase parental confidence in promoting healthy eating and body image in children and which kind of information resource, in what format, they would benefit from. The interview guide (translated to English) is available in the Supplementary Material.

Before the focus group interviews, participants were given the project information statement, written informed consent form, and demographic questions (age, education, marital status, sex). Due to COVID-19 restrictions, interviews were conducted *via* secure online services, except for one interview conducted in person. During the focus group interviews, one research staff member asked the group a question to facilitate group discussion until responses were exhausted before moving on to the next question. Two research staff members with graduate training were present during the interviews, with one serving mainly as an observer. All sessions were video-recorded and completed in ~90–120 min.

### Analysis of the Data

The interviews were transcribed and analyzed using interpretative phenomenological analysis (IPA), a method aimed at describing how a given person makes sense of a given phenomenon within a given context. IPA can be used with small focus groups when attention is given to helping facilitate the emergence of individual voices within the group through both group composition and group facilitation (51–53). Even though there are known challenges with extrapolating an individual's experience from a focus group (54), groups of peers can strengthen individual accounts (55) due to participants' similarities and peer-to-peer interactions. Our sample included two homogeneous samples with shared experiences on sensitive and stigmatizing topics such as overweight, unhealthy eating habits and poor body image. We organized the participants in small groups dividing parents and health professionals as IPA favors small samples with participants who have similar experiences, thus the parents' and health professionals' lived experience could be shown while accommodating similarities in the group (51). The small number of participants in the focus groups made us able to include all participants to voice their accounts, while writing notes about the dynamic, interactions, and positions in each group (56, 57). According to Githaiga (53) larger groups of 13 participants are difficult as participants are unable to talk in depth on their own accounts. Our groups were not larger than 6 participants, which enabled us to explore each individual account of the themes in a group setting. A further reason for keeping the groups small was that all but one were conducted online, where larger groups would have been difficult to manage.

The interviews were further facilitated to enhance the individual accounts within the group, avoiding the group becoming of “one voice” in the analysis (52). The researchers who performed the interviews had broad experience with leading group therapy, peer to peer groups, and conducting focus groups making them equipped to manage and modify the dynamic of the groups (e.g., making all participants share their experiences, avoiding some to dominate the group). We used the group dynamic from a focus group to enhance idiographic accounts with shared experiences of the sensitive topics explored in this study, which is close to the phenomenological epistemology of IPA (54).

The analysis was performed by three independent researchers, including the two present at the interviews. Each researcher performed an initial listening and reading/watching of the interviews, along with a thematic coding. This coding was refined through a second read-through of the transcripts and a group discussion. After that, the first author continued refining the themes, and all the authors discussed different interpretations. Similarities and deviations in the interpretation of the interviews and themes were discussed until a coherent interpretation was reached.

## Results and Themes

**Figure 1:**
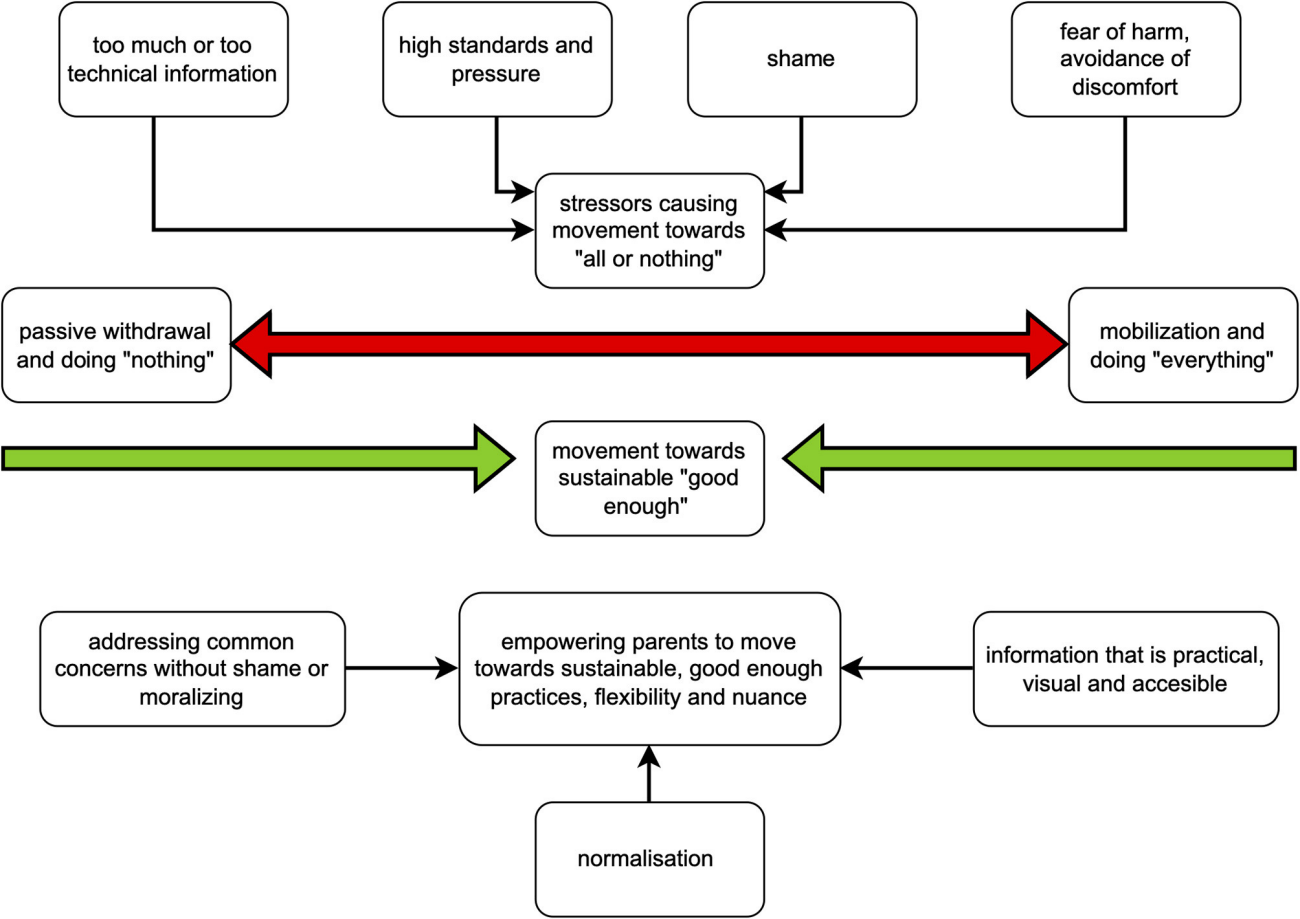
Summary of main results and suggestions (placeholder).

Overall, the interviews contained an overarching sense of parental stress and concerns related to a fear of “doing harm” to the children. This was found in both parents and professionals. The results conveyed that weight and body image are complex topics and that having a child who was classified as overweight was related to high levels of shame and a sense of having “failed as a parent”, further complicating interventions and follow-up. There were also indications of possible gender bias concerning weight and body image issues, with fathers and boys described as being less “visible” than their female counterparts. Interpretations of reoccurring themes and selected quotes from participants are described below. A presentation of salient and recurring themes is presented in Figure 1. The results from the interviews are broadly classified into themes related to (1) challenges and concerns and (2) possible solutions. Finally, perspectives on how to better approach weight and body image, as described by parents and professionals, are presented and interpreted in light of clinical theory on the effects of stress on adaptive behavior (27, 29) within an interpretative phenomenological framework.

### Challenges and Concerns

#### A Complex and Stressful Landscape

Parents found it demanding to keep current on recommendations on healthy eating, as expressed by one mother: “*Many people have more than enough to make the day… demanding to make the day add up... do not have the strength to be up to date on everything.”* In addition, parents described information on lifestyle as widespread in parental web forums and social media. “*A lot of the info I see comes from Facebook or forums and has a bunch of unauthorized opinionators. They do not have an education [and] do not understand when answering questions. It is scary and incredibly accessible*.” Particularly information on internet forums could contribute to negative parental stress. “*It is a hornet's nest. Whatever you do is wrong*.” Professionals confirmed the parental experiences of relating to a large amount of complex and diverse information. “*Think it is challenging. Lots of information, hard to trust what you know*.” Available and official informational resources for food and activity were described as overly technical and hard to grasp by professionals, leading to a further gap in knowledge among parents already knowledgeable and those with more challenges or less resources. As expressed by one of the professionals: “*The language can be very technical. Talk about training, minutes, intensity (…). The same with the diet. The diet also becomes difficult to follow.”* The professionals described how knowledge around healthy eating and food practices varied widely among parents they were in contact with in primary care, linking the variations to socio-economic class and health literacy. “*There is no shortage of information, but so many are not able to do it [change lifestyle]. Socio-economically skewed. How to reach the families who really need it the most… they are unable to make use of available information.”* Therefore, information was seen by the professionals as widely available for those who needed it the least and least available for those parents who needed it the most.

In addition to parents being exposed to a complex and sometimes confusing informational landscape related to food and weight, external pressures and standards were described as higher for parents now than for earlier generations. One parent expressed: “*There are so many external factors that come into play now, affecting children (…) do not think there were so many impulses before.”* Several parents spoke of pressures to be perfect, with a strong and prevalent sense of being judged by other parents or professionals. The stress related to perceived judgment was identified in all focus groups. Professional services and other parents were described as a source of stress. For example, stories of parents “training” their children for health check-ups, comparing their children's developmental progress to the progress of other children, seemingly competing over markers of “good parenthood” such as intricately packed lunches, were told by both professionals and parents. One parent stated: “*[I am] constantly worried about making mistakes, mistakes in the eyes of others, my own eyes, I feel constantly evaluated as a parent*.” Likewise, official guidelines on food and activity were seen as hard to meet for many parents: “*Parents see it as insurmountable.”* Follow-up around overweight was experienced as particularly stressful by both parents and professionals. “*I think a lot of what we do is experienced as an exam for many parents—both the weighing and measuring and in the cases where physiotherapists are involved.”*

#### Avoidance of Discomfort and Food as Regulation

According to professionals, parents providing children with food characterized as “comforting foods” (e.g., smoothie pouches) was increasingly frequent and interpreted as attempts to quickly still signs of discomfort or fuzziness in children. “*Something that has changed a lot…pouches of food—there are huge amounts, it has completely taken over. (…). I think the pouch is not there to supplement nutrition, but the child may be upset or impatient or scream, and then it [food] comes out, to distract, comfort, encourage, not for nutrition.”* Using food to stave off distress was interpreted by professionals as trying to meet emotional needs in some families: “*Parents who are not good enough at talking about emotions…‘eat the emotions'… for example, fuzzy children—parents regulate the child by feeding… finds that it works to give the child a bottle*.” This pattern was seen as starting at a very early age, often while nursing, with parents offering either breast or food as a distraction, entertainment, or comfort. As noted by one of the professionals: “*Food as comfort is another keyword*.”

#### A Narrow Normality

A sense of dichotomization and lack of flexibility in parental practices related to lifestyle habits was described. Parents told of a sense of a narrow normality regarding diet, weight, and activity. The professionals confirmed this observation. The missing sense of “normal” parental practices would often lead to self-doubt and uncertainty about what constitutes “good enough” practice. Self-doubts could include comparing own practices with “parenting ideals” with high standards like one mother expressed: “*What is good enough? If you are going on a hike after kindergarten, what is good enough? Do you have to go for a hike every weekend? Is it good enough to be in the garden or find a playground*?”

Some parents described themselves as being “above average health-conscious”, actively seeking out information. Some of them felt that official informational resources did not adequately describe the dangers of an unhealthy diet. As one parent expressed it: “*It is slightly under-communicated how much damage a very unhealthy diet can do. Maybe a little taboo*.” Some parents mentioned concerns related to sugar intake and less healthy foods served outside the home: “*We cannot enjoy ourselves as much at home because they have done it [had ice cream] in kindergarten*.” The behavior of families perceived as less health-conscious was also a worry: “*I am worried about those who are not worried. It is a big problem, those who are completely uncritical in all directions*.” On the other end of the spectrum, some of the participants considered themselves considerably less health-conscious, with a seemingly low concern regarding sugar intake, vegetable intake, or food quality, not wanting to exert any strict rules around eating habits: “*If you have eaten dinner you can get ice cream afterward. It should not be a very strict diet.”* In addition, some parents generally did not seek information on healthy eating or body image.

Professionals confirmed this dichotomy in parental practices. They often observed a lack of flexibility in parental practices, resulting in an “all-in or not-at-all” attitude related to lifestyle habits, as represented in Figure 1 as a movement toward the extremes of the continuum: “*The normal area has become a little smaller, it is so on the outer edges—there is a lot that is good enough…but it seems as if you do not get to the* ***optimum***, *you completely let go the other way.”* This all-or-nothing attitude was not limited to eating practices but included attitudes and behavioral practices related to physical activity. The official national recommendations were seen as conducive to this dichotomy. “*[The recommendations]are so* ***much***, *if they do not manage it completely, they [the parents] give up...[it] becomes all or nothing. Turns black and white*.”

#### Fear of Doing Harm

Fear of harming their children was described across all focus groups. Together with increasing stress, pressure, and a narrowing of normality, this fear was described as a cumulative negative spiral by both parents and professionals. Among all the focus groups, a common theme was a strong concern for children not eating enough food or being picky eaters. As a result, various strategies for making a child eat was the most sought after-information among the participants and the issue seen as the hardest to confront. Several parents told about adjusting meals or offering foods they did not see as healthy choices to have the child eat enough. All the professional focus groups confirmed a strong parental concern related to children eating enough food: “M*any are afraid that they [children] will get too little to eat.”* Professionals described parents going to considerable lengths to get children to eat what they thought was enough. The concern was not perceived to be around *what* the children ate, but the *amount*. One professional described: “*a mother who made five dinners every day (…). No fruits or vegetables for the girls, but that was not their concern. It was that everyone should get* ***enough***, *and that everyone would get something they liked. So, the concern was not whether it [dinner] was healthy. And they had two children with overweight.”* The parental fear of insufficient food intake was described as present very early in development. One professional saw challenges with breastfeeding as a possible cause: “*Perhaps struggling with breastfeeding can trigger a fear that children will not get enough food. Perhaps breastfeeding pressure can also be a gateway to unfortunate awareness of food. And a stressful food situation.”*

Many parents had unrealistic expectations to food intake and a lack of trust in the child's ability to regulate their own hunger and satiety, according to the professionals: “*Parents have somewhat unrealistic expectations of the amount they will eat. Trying to convey that the children have their own regulatory mechanisms in relation to appetite, they recover if they eat a little less a day or two.”* The lack of trust in the child's regulation and fear of children not getting enough food was seen as a risk factor for unhealthy and disturbed eating patterns: “*There is a risk of overeating (…) becomes thrown off right from the start*.” Such patterns of disturbed eating was seen as coming with possible negative consequences: “…*making children used to leaving the table feeling stuffed (…) perhaps taking that feeling with them for the rest of their lives.”* Parents reiterated the professionals' impression and found it difficult to trust the child's own ability to regulate their appetite: “*In theory, they say the child should control it [appetite] herself and be allowed to eat as much as she can, but when my child does not eat meal after meal (…) it is incredibly difficult.”* A conflict between disturbing and trusting the innate regulation of the child was expressed by many parents: “*Part of what is difficult about the child being a picky eater apart from if he gets enough food is the relationship with food… [I] want him to be able to regulate it [food intake]… that he does not eat because someone says that ‘now you have to eat'.”*

In addition to a fear of children not eating enough, parents were afraid that they would harm their children with their practices related to food, activity, and screen time. They were afraid to make the wrong choices or do too much or too little. “*My biggest concern is that I will harm the child in some way… I go weekly rounds with myself. Is it good or bad what I do? How much do I harm the child?*” Regulation of screentime was seen as universally challenging by both parents and professionals. Parents expressed fears that screens were so tempting that they would displace physical activity or play: “[It is] *so exciting inside with screens … it is more fun than being outside*.” However, limiting screentime was seen as difficult both because of its omnipresence in everyday life and fears that limiting access could cause social exclusion: “*The pressure is too great, you [the child] get excluded if you do not have ‘Fortnite', ‘Snap'….”* Professionals confirmed that screen time was becoming a large part of children's lives: “*Everyday-activity is a lot of time with a screen*.” Professionals expressed worry related to parental skills related to screen time: “…*they [parents] say that there is a lot of screen… as if it is impossible to change*.”

Parents also expressed worry about not making healthy enough choices or passing on bad habits to their children if they were not vigilant. As one parent expressed: “[*I am] worried that if we have a bad week when we are tired, and we choose ‘easy solutions'... that bad weeks with bad food will spread. And that the kid will be fat and lazy and not happy.”* A concern of passing on rigorous standards that were hard to meet was also expressed: “[*I am] also a little worried that we are overcompensating (…) we must be active every day and eat healthy (…) that it is not possible to have a lazy day.”*

#### Parental Concerns Around Children's Body Image

Body image was a highly challenging topic wrought with potential pitfalls and fear of harm described by parents and professionals. As one mother said: “*An overriding concern for me is that she will get a negative body image. In the long run, a disturbed relationship with food and activity*.” Some parents were concerned about body comments being possible forerunners to eating disorders. “*When it comes to things like my daughter [saying] ‘I have a big belly' (…), then I think ‘help… how can she think that... Oh, this is the first step toward an eating disorder'.”* Because of the fear of body comments leading to later body dissatisfaction and risk of development of ED, several parents told of difficulties responding to the child's appearance comments. “*[I am afraid] that we won't be able to say the right things or support a healthy self-image.”* Parents felt a big responsibility dealing with the child's feelings around body image. This was because they felt it could shape or break their children's future health and wellbeing both physically and mentally: “*It is a big responsibility (…)[you] can get seriously ill if you get a problem in one direction or another, if you get a problem with body image and food.”* The professionals mirrored the importance of a healthy body image: “*It is so important, so crucial to health. What do older people with health challenges struggle with? Self-image and body experiences- [they] have experienced not having mastered their body, not being fond of their body….think it is essential in life.”* Although body image was a topic both parents and professionals regarded as highly important and challenging, none were aware of any available resources they could use other than basic children's resources naming body parts or programs on puberty and sexuality.

#### The Shame of Overweight

Body image in children with overweight was deemed as particularly difficult for both professionals and parents. Having a child with overweight or obesity was described as a sore topic and difficult to approach in all groups. Feelings of shame related to having failed as a parent if the child was categorized as overweight were described by both parents and professionals, as expressed by one professional: “*Parents perceive the notification of the child's overweight as if they have failed, [they] will not talk about it in front of the child.”* This feeling of shame was recognized as detrimental for motivation by both professionals and parents and could make parents decline follow-up services from primary care. As expressed by one mother: “*For many, [it is]a very painful topic if someone points out that their child is overweight. And then they [parents] want no more to do with those who point it out.”* In addition, there was widespread concern amongst parents that conversations about food or weight could cause problems with body image or ED or vice versa: “*We do not want her to have a problem with food later because we stress about it [weight] now.”* Avoidance of these conversations made it challenging for parents to regulate food intake in children with larger appetites without referencing weight. Professionals confirmed that parents found receiving feedback regarding the child's weight status difficult, often needing separate consultations without the child being present, or avoiding the topic altogether. “*Many people are afraid to talk about it [weight] with their children, afraid to do anything about the child's self-image and how they think about themselves.”* Parents with personal histories of overweight, bullying, or ED were seen as particularly vulnerable in the follow-up of ChO, demanding greater flexibility and sensitivity from the professionals: “*It is completely wrong to talk about calories to someone who is eating disordered.”*

#### Stretched and Under-Resourced Professionals

Parallel to parents' increasingly demanding and stressful situations, professionals described their work situations as progressively complex and stretched due to a lack of resources and ever-increasing tasks. Professionals needed resources enabling them to work more preventatively around diet and weight problems: “*We should definitely do more prevention… now we are ‘putting out fires', all the time*.” They characterized their follow-up of parents, including follow-up of children's weight status, as under-resourced, with a “*sandwich list of topics to get through in little time*.” Professionals recognized overweight in children as particularly challenging and time-demanding because of its complexity. Most professionals perceived childhood obesity as grounded in more complex factors than nutrition and activity, such as family stressors (e.g., divorce, personal problems). “*In the vast majority of cases, it is much more than that they [children] eat too much and move too little, usually much more complex than that.”* Childhood obesity was also seen by many as steeped in negative emotions, making it challenging not to tread wrong. Professionals described a fear of jeopardizing the working alliance with parents: “*We have to get the parents involved, we have to do it a little carefully, with respect and ‘entice and trick' a little (…) very demanding.”* While the professionals deemed themselves knowledgeable in nutrition and healthy eating, they did not feel equipped or confident to have conversations about the complexity of weight and body image with parents or children. All expressed a need for more resources, both time and personnel and suitable interventions and methods. In addition, the professionals expressed a need for resources on dialogue skills and knowledge on boundary-setting to be able to provide support and recommendations related to weight and body image: “*How to be a good sparring partner (…) how to build people's self-image, that is what I feel we lack and we should have more of.” “More knowledge about how to set boundaries, how to talk to parents about difficult topics.”* Finally, the professionals pointed out that work with obesity and body image would benefit from more interdisciplinary work, including public health nurses, physiotherapists, psychologists, doctors, and nutritionists working together. “*If we could have more interdisciplinary consultations at the health center. It would be terrific.”*

Parents confirmed the notion of professional services being stretched on time and expressed a need for more resources and knowledge. Moreover, the children's follow-up consultations were described as “bare-boned” and packed with many health topics to get through in a minimum amount of time. However, the child and family health centers were also seen as an important influence and a source of near-universal reach for parents, regardless of parental interest and motivation. Child daycare centers were further described as very important for preventing ChO in terms of information, normalization, guidance, and reach.

#### Body Image—Forgotten Boys, Sidelined Fathers?

The parents expressed concern for girls' body image, adding that men and boys likely experienced less body pressure. As expressed by one mother: “*More worried about the girls than the boys (…) yes, maybe more body pressure when older, social media and such.”* In contrast, parents mainly conveyed stories of boys struggling with body shame and body-centered peer pressure. They conveyed stories of boys (<6) coming home from kindergarten with concerns about their height, muscularity, strength, speed, and weight and a culture of comparison and open discussion of bodily merits. “*He is a little sad sometimes because he is the smallest... if they [little boys] stand out on something, then they reflect and can get upset because they deviate from a kind of ideal.”* There were stories of boys demonstrating exercises for defined muscles, refusing food out of weight concerns, and wondering if they were faulty due to a small size relative to other children: “*He was the smallest in his kindergarten. [he] wondered if there might be something wrong with him*.”

In addition to seemingly lower concern regarding boys' body images than girls, the fathers' role was described differently from mothers' responsibilities. Mothers were seen as having a much more prominent role regarding food, weight, and body image, functioning as administrators of the family life and food choices, the bearers of knowledge, and gatekeepers of new information. “*In many families, the mother is the project leader*.” All groups agreed that engaging fathers was challenging but important for implementing new habits and practices in the family. Fathers were seen as important for children's body image and a possible source of body positivity, particularly for boys. One mother remarked: “*Men have a lot to contribute to children and body image. Where we as women look for faults, maybe men are a little better at praising their bodies?”* At the same time, fathers were described as less visible, sometimes almost sidelined, perhaps due to a perception that men knew less of body pressure and body image than females. The parents expressed that it would be necessary to explicitly aim resources at both parents to increase fathers' engagement, for instance, through webinars which parents could attend together or child-minding at events.

### Possible Solutions: What Do Parents and Professionals Want and Need to Approach Eating, Body Image, and Weight?

#### Normalizing Without Moralizing or Shaming

Information on diet and activity perceived as moralizing or fear-inducing was uniformly seen as ineffective and hurtful by both parents and professionals. Parents expressed that moralizing information induced shame and avoidance rather than readiness for dialogue or motivation for change. “*If someone comes and says [for instance] ‘your child has had three ice creams today, it's a* ***scandal***...'. ***[it's] easy to activate defense and soreness***.” In addition, moralizing information was seen as possibly pushing parents toward “echo chambers” supporting the parent's current beliefs rather than evidence-based practices. As one parent put it: “*if [information] is moralistic, people seek sources of information that support the way you choose to be a parent rather than what supports what is good for the child.”*

Professionals also warned that appearing too expert-like could shame and humiliate parents who were struggling. “*Do not appear as experts and look as if you are on top of everything… in training tights doing deep squats…and telling the parent group that ‘physical activity is so good… if you only get enough'. It is very humiliating to appear like an expert when meeting someone who is struggling.”* Professionals saw moralizing information as widespread in their line of work and particularly unhelpful for parents already stretched or struggling with lifestyle habits: “[*like professionals*] *coming to the parent meeting and saying ‘everyone* ***must*** *bring a packed lunch. Everyone* ***must*** *have breakfast before they arrive'.”*

A lack of cultural adaptation of resources for parents could create additional barriers for some families and cause unnecessary shaming related to, for instance, cultural differences in traditional food staples. As one professional expressed it: “*Many are proud of their culture. Take the pride away from them and force the ‘Norwegian' [the majority culture] over their heads and say that ‘this [whole meal bread] is much better than what you have'… it is not a good way to gain trust.”* Information that could be flexibly adjusted to different food cultures was seen as more inclusive and, therefore, more likely to succeed. “*Try to understand their culture (…). Inform so they can make choices and feel they can master it themselves*.”

Information that could normalize challenges and complexity was seen as both motivating and shame-reducing. As one parent expressed it: “*It would be better to start by normalizing… being understood in the situation you are in when you are struggling with something.”* Normalization included information on expected standards of healthy living. Several parents expressed a desire for advice on “good enough” practices, avoiding all-or nothingness: “*[It is] important to point out that it is not all or nothing- help people find the balance*.” The professionals confirmed that parents wanted and needed stories of normality and “good enough practices”. Parents spoke of normalization, which could take place within groups of parents, discussing common challenges: “*Have groups where you can talk about what is difficult (…) hear that things are common.”* One of the professionals stressed that normalizing information should not go against recommendations. As with shame, normalizing was suggested as an antidote for parents seeking alternate and less reliable sources of information, like forums: “*I think it is important to normalize the vast majority of parents and the vast majority of ways of raising children….”*

#### Empowering Parents Through Access to Easily Available, Visual, and Practical Information

The information and recommended practices should be experienced as manageable in terms of resources and time (i.e., moving toward a sustainable normality as seen in Figure 1), not necessitating perceived impossible standards like “*going out to shoot a deer… homegrown [ecological food], and all that*.” Parents and professionals agreed that interventions should be universally available for everyone but provide more to particularly vulnerable families. In terms of delivery, parents expressed a desire for a mixture of person-adapted face-to-face counseling centered on their situation, along with general and practical information in an app or on a website. Most, but not all, parents believed informational brochures were a thing of the past. Several parents mentioned apps and webinars as a form readily available for parents. It was also expressed that webinars could help bring more fathers aboard, creating more cohesive parenting practices: “*Being able to see it together with both parents would be very helpful.”* Webinars had the added benefit of allowing parents to unite on a topic: “*We can become more cohesive and understand the issues better.”*

Professionals stressed the importance of giving straightforward and accessible advice: “*It must be simple advice in relation to... what food, not too much advice, not too much professional language...”* In line with this, parents expressed a desire for easily available information, for instance, over social media, using a simple language accompanied with visuals, focusing on practical advice, followed by facts. “*Get it simple and visual. Self-help stuff [mimes showing practical examples] … have an easily linguistically accessible thing here. And then below it, there can be facts, justification.”* Advice that could help families make gradual steps toward better choices was seen as very important: “*Not either or... a gradual transition (…) take it gradually forward....”*

## Discussion

This study was part of a translation and cultural adaptation of the CBCC into a Norwegian setting. Interviews covering views and concerns around healthy eating, activity, and body image were conducted with three parental and three professional focus groups. The interviews identified challenges, concerns, and possible solutions to working with prevention of ChO and body dissatisfaction with parents. These points are discussed within a stress-informed perspective (27, 29, 49). Finally, the confident Body Confident Child intervention (CBCC) (19) is presented as an intervention that can meet all the major issues raised by parents and professionals in this study. See Figure 1 for a summary of the main results and primary suggestions.

Food, healthy eating, and body image was described as a complex and stress-inducing landscape by both parents and professionals. Results indicated that parents were faced with overly technical information from professional health services, alongside a steady flow of information of uncertain quality from social media, media, and other parents. A culture of comparison between parents and fear of judgment was challenging for both professionals and parents. Grappling with high parental standards and fear of not having “healthy enough” habits was mentioned by several of the parents. Further, parents were scared to “lose control” over healthy or unhealthy habits and therefore hurt the child's health, body image, or relationship with food. Parents saw the task of regulating eating and body image as a big responsibility with possible significant impacts on future mental and physical health. A reoccurring theme among parents was challenges with regulating food intake. For example, a common concern among the parents was fear of children not eating enough food, concerns over sugar intake, or making unhealthy choices in general. Another reoccurring theme was the uncertainty of meeting children's negative body comments. Parents were afraid to answer “the wrong thing” because they feared it could cause disordered eating patterns or induce low self-esteem. Challenges with emotional regulation was a reoccurring theme among professionals who noted an increasing tendency in parents trying to avoid signs of discomfort, fuzziness, or distress in their children, often using food as comfort and regulation. Overall, the results indicate that parenting, healthy eating, and body image is perceived as a stressful landscape fraught with concern by parents and professionals.

The ability to respond to possible harm to ourselves or our children is crucial for survival (27, 29, 58). *How* people react is influenced by personal history, individual and contextual resources, and perception of manageability (29, 49). When we perceive a situation as more manageable, we are more likely to access higher functions of reflection, deliberation, and regulation than when we perceive a situation as less manageable. Thus, higher perceived parental stress, higher vulnerability, or lower resources personally or contextually can contribute to less adaptive parental behaviors. An experience of more manageable stress one is sufficiently resourced for will likely lead to mobilizing parental behaviors actively engaging the situation to alleviate or reduce the perceived parental stress (29, 49). In terms of healthy living, this could include behaviors such as actively seeking information on and taking control over health behaviors such as sugar intake or physical activity or trying to influence the immediate environment (e.g., the family) to be healthier (moving along the “mobilization axis” seen in Figure 1).

If, however, a parent experiences oneself without sufficient resources to act, or a situation is seen as unmanageable, passive avoidance represents the second main line of defensive behaviors (Figure 1) (27, 29). The purpose of passive-avoidant behaviors is generally to passively withdraw oneself from a source of stress or threat by orienting away mentally and emotionally from something stressful. Regarding healthy living, passive avoidance could entail not seeking out information about dietary advice or physical activity, in addition to not engaging in or seemingly not being conscious of healthy behaviors (moving along the “passive withdrawal” axis in Figure 1).

In line with a hypothetical divide in active and passive parental behaviors, parents and professionals described a narrow parental normality and thus a tendency to think and act in extremes (all or nothing behaviors). Either families sought information actively and tried to live in a very health-conscious way in terms of sugar, food, and activity, or they were seemingly not concerned with healthy behaviors, perhaps because of the perceived high standards seen as necessary to live healthily *enough*. A socio-economic divide was mentioned by professionals noting a difference in health literacy, namely how families could “make use” of information about healthy living or not. In view of a stress-resource perspective (29), parents with fewer resources or more vulnerabilities may be more vulnerable for increased family stress and hence more passive, avoidant health behaviors. The added cost of healthy food compared to less healthy options (59) contributes to socio-economic divides in healthy living and family stress and must likely be addressed to help bridge socio-economic gaps in healthy living.

Parents who had children with weight challenges described a sense of profound failure and shame, making overweight and obesity a particularly sensitive, challenging, and stressful topic. The sense of parental shame related to child under- or overweight was confirmed by the professionals in our study. Of importance, shame evolved as a potent behavioral modifier aimed at increasing survival by keeping an individual in line with flock norms (60). The ability to feel some shame is, for that reason, necessary for social cohesion. However, feeling too much or unfounded shame is not. Because of the importance of shame and social acceptance for survival, feelings of shame and social rejection are regulated by some of the same brain areas as physical pain and fear (61–63). Situations that elicit shame can activate the same defenses as bodily harm (60, 64), particularly defensive withdrawal (65). It may, therefore, be vital to address parental shame related to a child's weight to avoid passive, avoidant health behaviors and responses to interventions.

Related to this, any intervention that inflicted or increased shame was deemed particularly detrimental for parent engagement and motivation, and even more so for families already struggling. Obesity interventions arousing parental shame were seen as contributing to challenging relationships between parents and helpers, complicating follow up or leading to dropout. Likewise, any information or intervention from primary care services perceived as moralizing or fear-inducing was uniformly seen as ineffective and hurtful, further contributing to parental shame and stress, resulting in avoidance and challenging interactions between parents and health professionals. The unfavorable effects of interventions invoking parental shame or fear are understandable within a stress-sensitive framework, as defensive responses generally limit the potential for reflection and connection (49, 58). This perspective has important implications for addressing and treating potentially sensitive topics like ChO. Specifically, clinical practice suggests that work with stressful or shameful topics necessitates adequately resourced professionals that can help parents regulate, reflect, and connect, not just deliver facts (49, 66). In essence, a focus on *co-regulation* can help make people more available for interventions. Unfortunately, parents were not alone in describing a sense of stress or a lack of resources. The professionals described the prevention and treatment of ChO as increasingly complex and stressful, making it hard to prevent health problems or to allocate the time or care needed for sensitive follow-up of parents and families. The parents confirmed the perception of a lack of time in primary care follow-up. Importantly, helpers stretched on time and resources are less able to meet others in an attuned way, making it hard to deal with complex or sensitive topics or emotions, reducing the quality of the help given (67, 68).

The combination of parental stress, feelings of shame connected with perceived “parental failure”, and under-resourced professionals may help explain why consultations related to ChO are demanding and why many parents decline follow-up related to weight challenges in Norway. Therefore, a way forward for successful preventative efforts may lie in interventions and contexts that properly resource both parents and professionals. Most importantly, interventions should avoid information or communication potentially inducing fear or shame, instead aiming to help parents explore, discover, and trust the follow-up. Further, parents may be better helped with communication emphasizing sustainable *good enough normality* around healthy eating, weight, and body image, as this might lower parental stress. As seen in Figure 1, this could mean helping parents at either end of the extremes (i.e., seemingly not trying, or trying to an unsustainable degree) move toward practices they can keep up in their day-to-day lives. Parents with personal histories of weight challenges, bullying, EDs, and fewer resources may benefit from closer follow-up delivered in a sensitive and co-regulating way. To achieve this, primary care professionals need sufficient resources to identify parents with extra needs and appropriately tend to those identified.

Appropriate interdisciplinarity may lighten the workload and increase the support system for both professionals and parents. In addition, interdisciplinarity increases the chances of seeing and addressing the complexity of weight and body image challenges. If professionals are given more time for preventative work avoiding purely reactive “repair work”, interventions may stand a better chance of success. Cultural adaptations and sensitivity are a further must to avoid the alienation of families from outside the majority culture. Our results also indicate that focusing on more than one caregiver and fathers (when applicable) and the importance of their individual contributions can increase family engagement and compliance with sustainable health behaviors. According to the parents, internet-based webinars, and services such as childcare in conjunction with interventions may help more than one adult in a family attend preventions or interventions. Finally, both parents and professionals desired practical, hands-on information on “good enough” and sustainable practices on both healthy living and body image. According to the results, such information should be given with evidence-based advice inducing trust, acceptance, and validation for parental difficulties.

Confident Body, Confident Child (CBCC) is an evidence-based preventative intervention for ChO, disturbed eating, and body dissatisfaction (19, 47, 48). CBCC provides professionals with practical, evidence-based interventions focused on easily accessible, practically oriented information around healthy living, body image, and common concerns in a non-moralizing way, empowering parents to make good choices. Importantly, CBCC addresses the concerns identified by parents and professionals in our study as particularly challenging and stressful. Perhaps the most important part of CBCC is its focus on creating a normalizing “good enough” family environment that allows children and families to relate to food and body image without fear or shame. For example, food is labeled as “every day” and “sometimes” foods based on their effects on health and wellbeing as opposed to dichotomous categories of food (e.g., “yes” or “no” food). Instead, CBCC teaches parents to encourage children to enjoy all food within responsible boundaries and in moderate quantities from an early age. This distinction creates and cultivates a family environment where no food is to be avoided or eaten because of or with shame. This is important as obesogenic food is everywhere, and restrictive parent eating practices increase the risk of over-eating (69) and possible escalation of overweight. In addition, CBCC addresses all the other major concerns identified by parents and professionals in our study. CBCC covers the child's natural regulation of appetite and food intake and provides information on health-promoting parental practices to regulate and set boundaries for food intake without inducing body shame or referencing weight. Parents are given strategies to help children listen to bodily signals of satiety and hunger and avoid snacking and grazing between meals. Further, CBCC includes strategies for parents to regulate highly palatable, low nutrient foods (e.g., snacks, sweets, comfort foods) in a family setting. CBCC also contains information on unfortunate practices to avoid and (why), such as food restriction. During the two CBCC sessions, the challenges with using food as regulation or comfort are addressed, which is important as the results indicated an increasing tendency for parental use of food as regulation. In addition, CBCC provides parents information on positive practices, such as repeated exposure to novel and healthy foods, positive social modeling of and positive reinforcement (e.g., praise) for healthy food choices, along with avoidance of pressure to eat as well as the importance of trusting the child's innate regulation to avoid “over-feeding”.

The prevention of body dissatisfaction, which can increase the risk of both ChO and ED, is a core focus in CBCC. The main principle is to promote acceptance and respect for body diversity and avoid appearance-based value judgments and comments. Actual examples of meeting appearance-focused body comments from the child or others (e.g., extended family or friends) are given to aid parents' ability to talk about such challenges with the child. In particular, CBCC provides parents with principles and examples of non-harmful body comments and examples of possible responses to comments, questions, concerns, or input around bodies or appearances from the child or others, highlighting the importance of not making appearance-focused comments to or around a child. Instead, CBCC teaches parents to start referencing themselves, the child, and others based on function, health, and self-care rather than appearance (including weight). Hence, the main principle is cultivating a focus on internal qualities, function, health, and joy of movement as a basis for self-and body image. Finally, CBCC also addresses the remaining parental and professional concerns, such as screen time and physical activity, and has resources for extended family members and online resources and material that can be studied in the home. In co-operation with trained personnel, CBCC can be implemented in child and family health centers and daycare, arenas identified as important and with far reach for parents.

Some adaptations have been made to the Norwegian version of CBCC based on this study. The changes are centered around the detrimental effects of parental shame, the culture of parent-comparison, and the need to normalize *healthy enough* habits. Specifically, content explicitly addressing parental shame was added, along with more plenary discussions centered on normalizing everyday parental experiences and frustrations related to healthy living and body image. In addition, we consider that it is vital to continue efforts to include and mobilize fathers to a larger degree and raise awareness of signs of poor body image in preschool boys.

The study has some limitations, particularly the relatively small, culturally homogenous sample of mostly females with higher education. However, the professionals interviewed had many years of experience working with a wide spectrum of parents, including highly resourced and more vulnerable families and families from culturally diverse backgrounds. It was a further limitation that all but one focus group had to be conducted online, as this limits the availability of non-verbal body language in the interview situation. However, video was available, allowing the capture of many non-verbal elements.

## Conclusion

As ChO and ED are simultaneously increasingly prevalent and hard to treat, there is a need for better ways to work preventively with ChO, disordered eating, and poor body image. Parents are the most important targets for interventions due to their essential role in shaping children's relationship with food and their bodies. This study conducted interviews with Norwegian parents and professionals on healthy living and body image as part of a cultural adaptation of the universal prevention program CBCC. Our results indicate that the parental pressure, expectations, and ideals surrounding healthy living, body image, and parenting today may be perceived as overwhelming for some parents, potentially influencing parental behaviors negatively toward dichotomous practices related to health behaviors and complicating dialogue and follow-up. A Norwegian cultural adaption of CBCC was made with extra emphasis on parental shame and normalization of parental experiences in a group format.

## Data Availability Statement

The datasets presented in this article are not readily available because the dataset was not approved for sharing due to its identifiable nature (qualitative data). Requests to access the datasets should be directed to charlotte.fiskum@ntnu.no.

## Ethics Statement

The studies involving human participants were reviewed and approved by Norwegian Center for Research Data. The patients/participants provided their written informed consent to participate in this study.

## Author Contributions

CF wrote the first drafts of the manuscript, prepared the submission, transcribed and translated the interviews, and took main responsibility for the data-analysis. ÅR contributed to initial planning and recruitment and data-collection, participated in data-analysis, and gave critical comments on drafts of the manuscript. TE-N was PI for the study and procured funding, took main responsibility for initial planning and recruitment and data-collection, participated in data-analysis, and gave critical comments and input on drafts of the manuscript. All authors contributed to the article and approved the submitted version.

## Funding

The project was funded by a grant from the Norwegian Occupational Therapy Association.

## Conflict of Interest

The authors declare that the research was conducted in the absence of any commercial or financial relationships that could be construed as a potential conflict of interest.

## Publisher's Note

All claims expressed in this article are solely those of the authors and do not necessarily represent those of their affiliated organizations, or those of the publisher, the editors and the reviewers. Any product that may be evaluated in this article, or claim that may be made by its manufacturer, is not guaranteed or endorsed by the publisher.
