# Supplementary material for: Prevention of Unhealthy Weight, Disordered Eating, and Poor Body Image in Children. Perspectives From Norwegian Parents and Healthcare Professionals
Source: Front Psychiatry. 2022 Apr 27;13:895781. doi: 10.3389/fpsyt.2022.895781 (PMC9094704; doi:10.3389/fpsyt.2022.895781)
Supplement: Supplementary file 1 [file Data_Sheet_1.PDF]

Interview- guide (Translated from Norwegian to English, Norwegian guide available upon request)

Semi-structured focus group interview

Questions to lead the focus group discussion with parents

1. What does 'healthy food' in children mean to you? (We would like to put the spotlight on children aged 2-6 years throughout this session).

2. What about 'body image'?

3. (Give definition of body image to parents: the thoughts and feelings around the body and appearance) When do you think children develop their own body image?

4. Are you aware of / use of resources for parents that provide information on how to encourage healthy food or physical activity in children?

5. Are you aware of / use resources that are designed for parents that provide information on how to encourage a positive body image in children?

6. What do you dislike about these resources, or how parenting information is currently being provided?

7. What gaps do you think there are in today's resources / information for parents? What do you want to see developed to promote healthy food and a positive body image in your child?

8. Is there anything else you feel will help increase your confidence in promoting healthy eating habits, physical activity and a positive body image in your child?

9. If new information / resources for parents were developed, what do you think would be the best time for parents to receive this information (e.g. As soon as you become a parent? When the child gets older? At what age?)

10. What would be best if new information / new resources for parents were developed? (e.g. information sheet, website, training course, contact of a professional)

a. Do you want to use / prefer a website with information on how to promote a healthy / safe body image, eating and physical activity in children?

b. Do you want to use / prefer brochures / booklets?

11. What must this package include to help you access and use the information (e.g. clear language, stories from other parents, videos, professional advice, evidence-based research)

12. Are there any other things you would wish to say, including worries and concerns as parents regarding what we have discussed?

Questions to lead the focus group discussion with health professionals

1. What does 'healthy food' in children mean to you? (We would like to put the spotlight on children aged 2-6 years throughout this session).

2. What about 'body image'?

(Give definition of body image: the thoughts and feelings around the body and appearance). When do you think children develop their own body image?

3. What resources are designed for parents that provide information about healthy food or physical activity in children, are you aware of / have you suggested to parents? (e.g. websites, brochures, books, courses and organizations)

4. What resources are there for parents about body image in children in Norway?

5. Do you mention or recommend these resources to parents? (e.g. through conversations during agreements or distribution of information sheets).

Concern among parents

1. What are some common concerns that parents discuss with you about their (preschool) children regarding:

a. to eat healthy

b. body image

2. At what point in the child's life do parents become more focused on or concerned about the child's healthy eating habits and body image? (interviewer comment: If professionals think that these appear at different times, ask at what age each comes)

Design of a course package (aimed at both parents and health professionals)

1. What kind of information would you suggest we include in a course to address parents' concerns about healthy eating habits and body image? (interviewer comment: If professionals think that these should be addressed separately instead of in combination, ask how each should be addressed)

2. How do you think this information can best be given to parents?

3. What do you think is not effective when working with parents (what should we avoid)?
